# Association of the Dietary Index for Gut Microbiota and Cardiovascular‐Kidney‐Metabolic Syndrome: The Mediation Effect of Phenotypic and Biological Age Acceleration, BMI, and BRI

**DOI:** 10.1002/fsn3.70815

**Published:** 2025-09-07

**Authors:** Dongdong Yu, Ting Cheng, Yuxuan Shi, Xingying Qiu, Geng Li, Yue Yang, Li Zhou, Zehuai Wen

**Affiliations:** ^1^ Second Clinical College of Guangzhou University of Chinese Medicine Guangzhou China; ^2^ First Clinical College of Anhui University of Chinese Medicine Hefei China; ^3^ First Affiliated Hospital of Anhui University of Chinese Medicine Hefei China; ^4^ Center for Clinical Research of Guangdong Provincial Hospital of Chinese Medicine (Second Affiliated Hospital of Guangzhou University of Chinese Medicine) Guangzhou China; ^5^ Science and Technology Innovation Center of Guangzhou University of Chinese Medicine Guangzhou China

**Keywords:** aging, cardiovascular‐kidney‐metabolic, dietary index for gut microbiota, NHANES, obesity

## Abstract

The relationship between gut microbiota, diet, and cardiovascular‐kidney‐metabolic (CKM) health has attracted attention. However, the relationship between the dietary index for gut microbiota (DI‐GM) and CKM syndrome has not yet been studied. Patients diagnosed with CKM syndrome from the NHANES 2007–2018 data were included. Dietary recall data were used to calculate DI‐GM. Restricted cubic splines (RCS) were employed to explore nonlinear relationships and determine the threshold for DI‐GM. The relationship between DI‐GM and CKM syndrome was analyzed using weighted logistic regression. Further, potential mediating roles of phenotype age acceleration (PAA), biological age acceleration (BAA), body mass index (BMI), and body roundness index (BRI) were explored. Sensitivity analysis using inverse probability of treatment weighting (IPTW) was also conducted. A total of 7252 participants were included, with the high DI‐GM group as the reference. In the crude model, the risk of CKM syndrome in the low DI‐GM group was significantly higher (OR = 1.27, 95% CI = 1.09, 1.49, *p* < 0.05). This association remained significant in the fully adjusted model (OR = 1.34, 95% CI = 1.10, 1.64, *p* < 0.05). The results of the IPTW analysis were consistent with the above findings. In the association between DI‐GM and CKM syndrome, significant mediating effects were observed for PAA (mediated proportion (MP): 14.84%, *p* < 0.001), BAA (MP: 21.45%, *p* < 0.001), BMI (MP: 24.17%, *p* < 0.001), and BRI (MP: 35.85%, *p* < 0.001). Low DI‐GM is associated with an increased prevalence of CKM syndrome. PAA, BAA, BRI, and BMI significantly mediate the relationship between DI‐GM and CKM syndrome.

## Graphical abstract

The interplay between gut microbiota, obesity, and CKM still warrants further investigation. Low dietary index for gut microbiota (DI‐GM) is associated with an increased prevalence of cardiovascular‐kidney‐metabolic (CKM) syndrome. Phenotype age acceleration, biological age acceleration, body mass index, and body roundness index significantly mediate the relationship between DI‐GM and CKM syndrome.

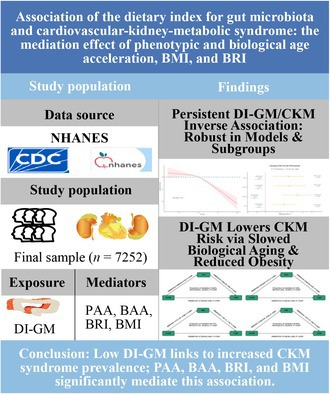

## 1 Introduction

As the understanding of the pathophysiological interactions between obesity, diabetes, chronic kidney disease (CKD), and cardiovascular disease (CVD) deepened, the American Heart Association introduced the concept of cardiovascular‐kidney‐metabolic (CKM) syndrome (Ndumele, Neeland, et al. 2023). CKM syndrome includes individuals with CVD risk due to the presence of metabolic risk factors, CKD, or both, as well as individuals with existing CVD that may be associated with or complicated by metabolic risk factors or CKD (Ndumele, Rangaswami, et al. 2023). Nearly 90% of U.S. adults meet the criteria for CKM syndrome, with 15% meeting the criteria for advanced stages (Aggarwal et al. 2024). This syndrome can lead to premature mortality, high morbidity, multi‐organ diseases, and high healthcare expenditures, primarily driven by the burden of cardiovascular disease (CVD). Age‐stratified analysis shows that the burden of CKM syndrome increases with age (Trimarco et al. 2024). Severe stages of CKM are associated with an increased risk of all‐cause and CVD mortality (Cao et al. 2025; Li, Li, et al. 2024).

The gut microbiota and its derived metabolites have become an important and key factor in the pathogenesis of metabolic‐related diseases such as chronic kidney disease, diabetes, and CVD (Datta et al. 2024; Li, Stražar, et al. 2024; Mansour et al. 2024; Zhang, Lv, et al. 2024). Dysbiosis activates inflammatory pathways, releases inflammatory factors, and contributes to oxidative stress and metabolic disorders, playing a role in the initiation, development, and progression of CVD and CKD (Cui et al. 2024; Dicks 2024; Dinakis et al. 2024; Lu et al. 2024; McCarthy et al. 2024; Niu et al. 2024; Vallianou et al. 2024). Dietary components with cardioprotective effects, such as probiotics, prebiotics, monounsaturated fatty acids, polyunsaturated fatty acids, carotenoids, and polyphenols, can regulate the gut microbiota and, in turn, influence cardiovascular health (Cuervo et al. 2024; Ghanbari et al. 2024; Khan et al. 2024). Kase et al. developed a new gut microbiota diet index (DI‐GM) to assess dietary quality associated with maintaining a healthy gut microbiota (Kase et al. 2024). However, studies exploring the relationship between DI‐GM and CKM are still lacking.

Aging is also considered one of the most important risk factors for CVD (Li, Jiang, et al. 2024). Phenotypic age (PA) and biological age (BA), newly developed epigenetic biomarkers of aging, provide more precise information on individual aging speed and offer insights into aging (Levine et al. 2018). Phenotypic age acceleration (PAA) and PA are significantly associated with all‐cause and cause‐specific mortality, helping to identify high‐risk individuals for multiple diseases and causes of death (Levine et al. 2018; Liu et al. 2018). Mao R et al. reported a significant correlation between the increase in biological age acceleration (BAA) and deleterious changes in cardiac structure (Mao et al. 2024). Mendelian randomization studies have confirmed that specific microbiota may have a causal impact on PAA (Xu et al. 2024). The microbiome in atherosclerotic cardiovascular disease (ACVD) is positively correlated with age (Dong et al. 2023). Some studies have confirmed that higher DI‐GM scores are associated with a lower risk of accelerated aging (An et al. 2024). However, the role of aging in the relationship between DI‐GM and CKM syndrome has not been established.

Obesity and cardiovascular metabolic diseases are major public health issues associated with changes in the gut microbiota (de la Cuesta‐Zuluaga et al. 2023). The relationship between gut microbiota and obesity remains to be determined (Singer‐Englar et al. 2019). Visceral obesity is considered one of the main risk factors for cardiovascular events and all‐cause mortality. The body roundness index (BRI), an obesity‐related indicator reflecting visceral fat distribution, has a U‐shaped association with all‐cause mortality (Thomas et al. 2013; Zhang, Ma, et al. 2024). Body mass index (BMI) and triglyceride glucose BMI (TyG‐BMI) have been identified as effective predictors for CVD and CKM syndrome (Li, Shen, et al. 2024; Zhang, Zheng, et al. 2024). Therefore, the interplay between gut microbiota, obesity, and CKM still warrants further investigation. Thus, this study aims to explore the association between DI‐GM and CKM syndrome by analyzing adult data from the National Health and Nutrition Examination Survey (NHANES) and investigating the potential mediating roles of PAA, BAA, BMI, and BRI.

## 2 Methods

### 2.1 Data Sources

The NHANES is a large, nationally representative, publicly available database conducted by the National Center for Health Statistics. Based on a complex sampling design, it is widely used in health, nutrition, and disease‐related research (Liu et al. 2018). The Institutional Review Board of the National Center for Health Statistics has approved the continuous conduct of the study, and all participants have provided informed consent. The data used in this study have been de‐identified and are publicly accessible (https://wwwn.cdc.gov/nchs/nhanes /Default.aspx).

### 2.2 Study Population

**FIGURE 1:**
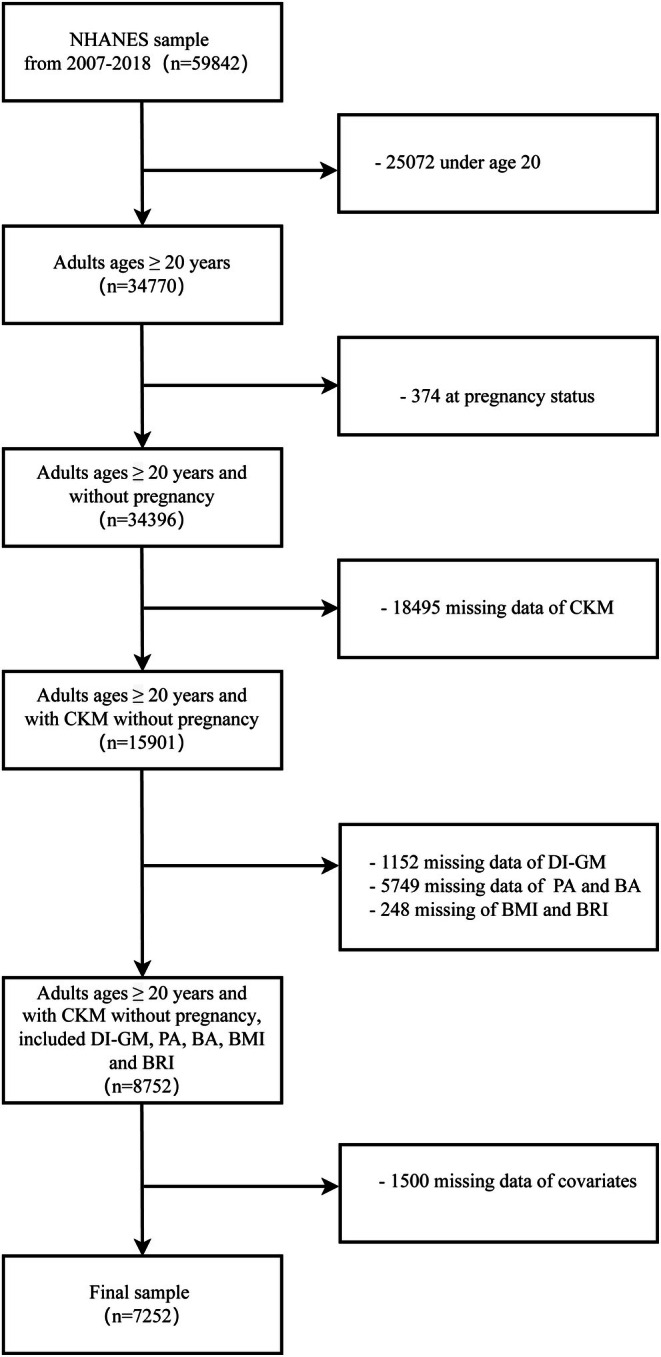
Flowchart of the screening process. NHANES, National Health and Nutrition Examination Survey; PA, phenotypic age; BA, biological age; DI‐GM, dietary index for gut microbiota; BMI, body mass index; BRI, body roundness index; CKM, cardiovascular‐kidney‐metabolic syndrome.

A total of 59,842 individuals were identified from the NHANES 2007–2018 data, and those who met the definition of CKM syndrome were included in the study population. The exclusion criteria for this study were as follows: (1) age under 20 years; pregnant women; (2) lack of CKM data; (3) lack of DI‐GM components; missing phenotypic age, biological age, BMI, and BRI; and (4) individuals missing other covariates. Ultimately, 7252 eligible participants were included in the analysis, as shown in Figure 1.

### 2.3 Definition of DI‐GM

DI‐GM was calculated based on the dietary recall data from the NHANES database, with its components and scoring criteria referenced from the study by Kase et al. (2024). The DI‐GM includes 14 foods or nutrients identified as components: fermented dairy, chickpeas, soybeans, whole grains, fiber, cranberries, avocados, broccoli, coffee, and green tea as beneficial components, and red meat, processed meat, refined grains, and high‐fat diet as unfavorable components. For beneficial components to the gut microbiota, a score of 1 is given when the intake is greater than or equal to the gender‐specific median; otherwise, a score of 0 is given. For unfavorable components to the gut microbiota, a score of 0 is given when the intake is greater than or equal to the gender‐specific median or when the proportion of energy from a high‐fat diet is ≥ 40%; otherwise, a score of 1 is given. Finally, the individual scores are summed to obtain the overall DI‐GM score. Green tea was excluded from the DI‐GM calculation due to lack of specific consumption data in NHANES, consistent with Kase et al.'s implementation for this dataset (Kase et al. 2024). The final DI‐GM included 13 components (range: 0–13) (refer to Supplement 1‐Supplementary Methods).

### 2.4 Definition of the Main Outcome

The staging structure of CKM syndrome ranges from 0 (no risk factors) to 4 (diagnosed cardiovascular disease, CVD) (Ndumele, Rangaswami, et al. 2023). We referred to the method by Aggarwal et al. to define CKM syndrome patients in the NHANES (Aggarwal et al. 2024): Stage 0, no CKM risk factors; Stage 1, severe obesity or dysfunctional obesity; Stage 2, metabolic risk factors or moderate to high‐risk chronic kidney disease (CKD); Stage 3, subclinical CVD or risk equivalents of CKM syndrome; Stage 4, clinical CVD of CKM syndrome. For comprehensive details regarding the assessment of CKM syndrome stages, refer to Supplementary Methods in Supplement 1. In this study, stages 2 to 4 were defined as the presence of CKM.

### 2.5 Definition of PAA, BAA, BMI, and BRI

PA and BA both reflect an individual's physiological health status and the deviation from their chronological age by integrating a series of biomarkers (Comfort 1969). PAA and BAA refer to the residuals generated from a series of regression models when phenotypic age and biological age are regressed against chronological age. If the result is positive, it indicates accelerated aging; if negative, it suggests slowed aging (Li, Wu, et al. 2024). PA calculation involves white blood cell count, lymphocyte proportion, mean cell volume, red cell distribution width, albumin, alkaline phosphatase, creatinine, glucose, and C‐reactive protein (hs‐CRP after 2015). BA calculation involves systolic blood pressure, albumin, alkaline phosphatase, blood urea nitrogen, creatinine, C‐reactive protein, glycated hemoglobin, and total cholesterol. The above are calculated using the “BioAge” package, with detailed formulas provided in Supplement 1 Supplementary Methods (Kwon and Belsky 2021; Li, Wang, et al. 2024). BMI and BRI were calculated based on waist circumference (cm), height (m), and weight (kg) measured by the mobile health screening center (Thomas et al. 2013).

$$\mathrm{BMI}=\frac{\text{Weight}\;\left(\mathrm{kg}\right)}{\text{Height}\;{\left(m\right)}^{2}}$$

BRI=364.2−365.5*1−Waist Circumferencecm2π20.5*Heightcm2

### 2.6 Covariates

Based on published research and clinical judgment, several potential confounding variables were considered, including age, gender, race, marital status, education level, PIR, smoking status, and alcohol consumption status (An et al. 2024). Racial categories include Mexican American, non‐Hispanic Black, non‐Hispanic White, other Hispanic, and others. Marital status is categorized as having a partner or being single. Education level is categorized as high school or less, high school or equivalent, and more than high school. PIR is a continuous variable. Self‐reported smoking and alcohol consumption statuses are binary variables, with “no” defined as having never smoked or drank, and “yes” defined as having ever smoked or drunk. Participants with < 100 lifetime cigarettes were classified as nonsmokers (“no”); all others as smokers (“yes”, including former/current). Individuals reporting < 12 alcoholic drinks in their lifetime were classified as nondrinkers (“no”); other respondents (including former/current drinkers) as drinkers (“yes”).

### 2.7 Statistical Analysis

According to the NHANES analysis guidelines, analyses accounted for NHANES complex survey design by applying sampling weights, stratification, and clustering through the R survey package (https://wwwn.cdc.gov/nchs/nhanes/tutorials/weighting.aspx). Consistent with the methodology described by Kase et al. (2024), the DI‐GM was initially constructed without applying sample weights during component scoring; however, all subsequent statistical analyses incorporated appropriate weighting procedures. First, we described the characteristics associated with CKM syndrome. Continuous variables, analyzed with independent samples t‐tests (which are robust to non‐normality given large group sample sizes; *n* > 2000 per group), were presented as means ± standard error (SE); categorical variables, assessed using Chi‐square tests, were presented as frequencies and percentages (%). Sensitivity analyses with nonparametric tests produced concordant results for the continuous variables (Supplement 2 Table S1).

We used univariate weighted logistic regression to analyze the relationship between each variable and CKM syndrome (Supplement 2 Table S2). Restricted cubic splines (RCS) with three knots were used to fit the potential nonlinear relationship between DI‐GM and CKM syndrome, and the cutoff value for DI‐GM was determined, categorizing it into a binary variable. Multivariate weighted logistic regression models were used to analyze the association between DI‐GM and CKM syndrome. Covariates were selected a priori based on clinical relevance and univariate screening (Supplement 2 Table S2). Race/ethnicity was excluded from models due to nonsignificance and lack of effect modification. The crude model without adjustment for any covariates; Model 1 was adjusted for age, gender, marital status, and education level; Model 2 was adjusted for age, gender, marital status, education level, smoking status, alcohol status, BMI, and BRI; Model 3 was adjusted for age, gender, marital status, education level, smoking status, alcohol status, BMI, BRI, PAA, and BAA. To further eliminate bias and control for baseline potential confounders between groups, we conducted a sensitivity analysis using inverse probability of treatment weighting (IPTW). As suggested by Lenis et al., survey weights were also considered in the IPTW analysis (Lenis et al. 2019). Furthermore, we performed stratified analyses to assess the robustness of DI‐GM.

Moreover, we further explored the potential mediating effects of phenotypic age, biological age, BMI, and BRI on the association between DI‐GM and CKM syndrome. Mediation analysis was conducted using the Sobel test, Bootstrap, and quasi‐Bayesian Monte Carlo methods, with 1000 simulations based on normal approximation.

All analyses were performed using R (4.4.0) software. Survey sample analysis was conducted using the “survey” package (4.4–2), and mediation analysis was performed using the “mediation” package (4.5.0). Phenotypic age and biological age were calculated using the “BioAge” package (0.1.0). Statistical significance was defined as a two‐tailed *p* < 0.05. Figure S1 shows the statistical analysis workflow.

## 3 Results

### 3.1 Characteristics of the Study Population

A total of 7252 participants were included (Figure 1), of which 5210 were diagnosed with CKM syndrome. The average age of all participants was 47.8 years. Notably, CKM syndrome patients were more likely to be older, male, married, have lower income and education levels, a history of smoking, and higher PA, BMI, and BRI (Table 1).

**TABLE 1:** Characteristics of the study population.

Variable	Total	Non‐CKM (*n* = 2042)	CKM (*n* = 5210)	*p*
Age^a^	47.78 ± 0.37	38.53 ± 0.51	52.25 ± 0.41	< 0.001
Sex				< 0.001
Male	49.0 (45.0, 53.0)	42.2 (39.2, 45.1)	52.3 (50.6, 54.0)	
Female	51.0 (47.3, 54.8)	57.8 (54.9, 60.8)	47.7 (46.0, 49.4)	
BMI^a^	28.99 ± 0.15	26.28 ± 0.22	30.30 ± 0.18	< 0.001
BRI^a^	5.37 ± 0.05	4.26 ± 0.07	5.90 ± 0.06	< 0.001
Race/Ethnicity^b^				0.930
Non‐Hispanic White	69.7 (61.8, 77.5)	69.1 (65.7, 72.5)	69.9 (66.2, 73.6)	
Non‐Hispanic Black	9.8 (8.3, 11.3)	9.9 (8.1, 11.7)	9.7 (7.8, 11.6)	
Mexican American	8.3 (6.5, 10.1)	8.3 (6.0, 10.7)	8.3 (6.3, 10.2)	
Other	12.3 (11.0, 13.6)	12.6 (11.0, 14.3)	12.1 (10.2, 14.0)	
Marital status^b^				0.010
Couple	64.3 (58.3, 70.2)	60.7 (57.5, 63.8)	66.0 (63.6, 68.4)	
Alone	35.7 (33.3, 38.2)	39.3 (36.2, 42.5)	34.0 (31.6, 36.4)	
Education^b^				< 0.001
Less than high school	4.6 (3.8, 5.5)	2.6 (1.8, 3.4)	5.6 (4.6, 6.6)	
High school or equivalent	49.0 (44.2, 53.7)	44.0 (40.4, 47.6)	51.4 (48.4, 54.4)	
College or above	46.4 (41.9, 50.9)	53.4 (49.7, 57.2)	43.0 (39.6, 46.4)	
Smoke^b^				< 0.001
No	53.6 (49.6, 57.7)	60.1 (56.4, 63.9)	50.5 (48.6, 52.4)	
Yes	46.4 (42.2, 50.5)	39.9 (36.1, 43.6)	49.5 (47.6, 51.4)	
Alcohol^b^				0.020
No	10.0 (8.6, 11.5)	8.2 (6.3, 10.1)	10.9 (9.6, 12.3)	
Yes	90.0 (83.4, 96.6)	91.8 (89.9, 93.7)	89.1 (87.7, 90.4)	
PIR^a^	3.07 ± 0.04	3.14 ± 0.06	3.04 ± 0.05	0.190
PA^a^	40.20 ± 0.39	28.83 ± 0.62	45.70 ± 0.45	< 0.001
BA^a^	25.85 ± 0.56	14.82 ± 0.93	31.18 ± 0.59	< 0.001
HEI‐2020	52.96 ± 0.38	53.41 ± 0.51	52.74 ± 0.41	0.18
Activity				< 0.001
No	19.7 (17.6, 21.7)	14.0 (11.68, 16.26)	22.5 (20.5, 24.6)	
Yes	80.1 (73.8, 86.4)	86.0 (83.7, 88.3)	77.5 (75.4, 79.5)	
Antdiabetic				< 0.001
No	90.8 (84.0, 97.5)	NA	86.4 (84.9, 87.9)	
Yes	9.1 (7.9, 10.3)	NA	13.5 (12.1, 15.1)	
Antihypertensive				< 0.001
No	94.4 (87.6, 97.3)	NA	91.9 (90.7, 93.1)	
Yes	5.8 (4.6, 6.4)	NA	8.1 (6.9, 9.3)	

Abbreviations: BA, biological age; BMI, body mass index; BRI, body roundness index; CKM, cardiovascular‐kidney‐metabolic syndrome; NA, not applicable, not presented, statistical instability from extreme weighted values; PA, phenotypic age; PIR, poverty income ratio.

^a^ Reported as mean ± standard errors (SE).

^b^ Reported as percentage with a 95% confidence interval (CI).

### 3.2 Associations of the DI‐GM With CKM

**FIGURE 2:**
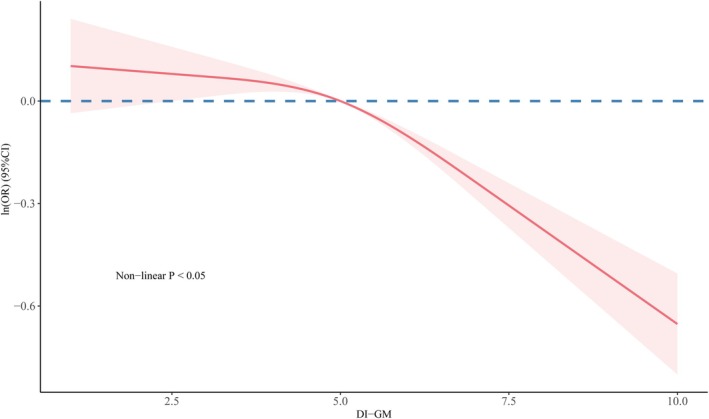
The RCS results between DI‐GM and CKM syndrome. DI‐GM, dietary index for gut microbiota.

As shown in Table 2 and Figure S2, each one‐point increase in the DI‐GM score was associated with an 8% reduction in the prevalence of CKM syndrome (OR = 0.92, 95% CI = 0.88, 0.96). Based on the univariate regression results (Supplement 2‐Table S1), we progressively adjusted the models using stepwise regression. The association remained statistically significant in the fully adjusted model (OR = 0.92, 95% CI = 0.87, 0.97). Furthermore, RCS analysis revealed a nonlinear relationship between DI‐GM and CKM syndrome, with the RCS model demonstrating superior goodness‐of‐fit compared to the logistic regression model (*p* < 0.05). Based on the RCS model, DI‐GM was categorized into high and low groups (Figure 2).

**TABLE 2:** Associations between the DI‐GM and CKM syndrome using the weighted multivariable regression.

Character	Crude model		Model 1		Model 2		Model 3	
	OR (95% CI)	*p*	OR (95% CI)	*p*	OR (95% CI)	*p*	OR (95% CI)	*p*
DI‐GM	0.92 (0.88, 0.96)	< 0.001	0.87 (0.83, 0.92)	< 0.001	0.91 (0.87, 0.96)	< 0.001	0.92 (0.87, 0.97)	0.002
DI‐GM category								
Higher DI‐GM	ref		ref		ref		ref	
Lower DI‐GM	1.27 (1.09, 1.49)	0.003	1.50 (1.24, 1.82)	< 0.001	1.36 (1.11, 1.67)	0.003	1.34 (1.10, 1.64)	0.010

*Note:* Crude model: adjusted for none; ref.: reference level/category. Model 1: adjusted for age, sex, marital status, and education. Model 2: adjusted for age, sex, marital status, education, smoking, alcohol, BMI, and BRI. Model 3: adjusted for age, sex, marital status, education, smoking, alcohol, BMI, BRI, PAA, and BAA.

Abbreviations: BAA, biological age acceleration; BMI, body mass index; BRI, body roundness index; CI, confidence interval; DI‐GM, dietary index for gut microbiota; OR, odds ratio; PAA, phenotypic age acceleration; PIR, poverty income ratio.

Using the higher DI‐GM group as the reference (Table 2 and Figure S2), the crude model showed a significantly higher risk of CKM syndrome in the lower DI‐GM group (OR = 1.27, 95% CI = 1.09, 1.49). This association persisted in the fully adjusted model (Model 3: OR = 1.34, 95% CI = 1.10, 1.64).

Sensitivity analysis incorporating race as an additional covariate revealed that the effect estimates for DI‐GM exhibited no substantial change, and its statistical significance remained consistent (Table S3), regardless of whether DI‐GM was modeled as a continuous or categorical variable. Further subgroup analyses (Table S4) demonstrated that lower DI‐GM levels were significantly associated with an increased risk of CKM in most subgroups (*p* < 0.05). Notably elevated CKM risk associations were observed for: males (OR = 1.44; 95% CI = 1.06, 1.95; *p* = 0.020), couple individuals (OR = 1.37; 95% CI = 1.05, 1.77; *p* = 0.021), college‐educated participants (OR = 1.37; 95% CI = 1.05, 1.78; *p* = 0.020). Stronger effects were found among: nonsmokers (OR = 1.45; 95% CI = 1.12, 1.90; *p* = 0.007), alcohol consumers (OR = 1.35; 95% CI = 1.10, 1.65; *p* = 0.005). The absence of significant interaction effects (all *p*‐for‐interaction > 0.05) indicated that the protective effect of higher DI‐GM levels was broadly consistent across all subgroups analyzed. Sensitivity analysis using IPTW further confirmed the robustness of this association in the fully adjusted model (OR = 1.15; 95% CI = 1.01, 1.33; Table S5).

### 3.3 Mediation Analysis

**FIGURE 3:**
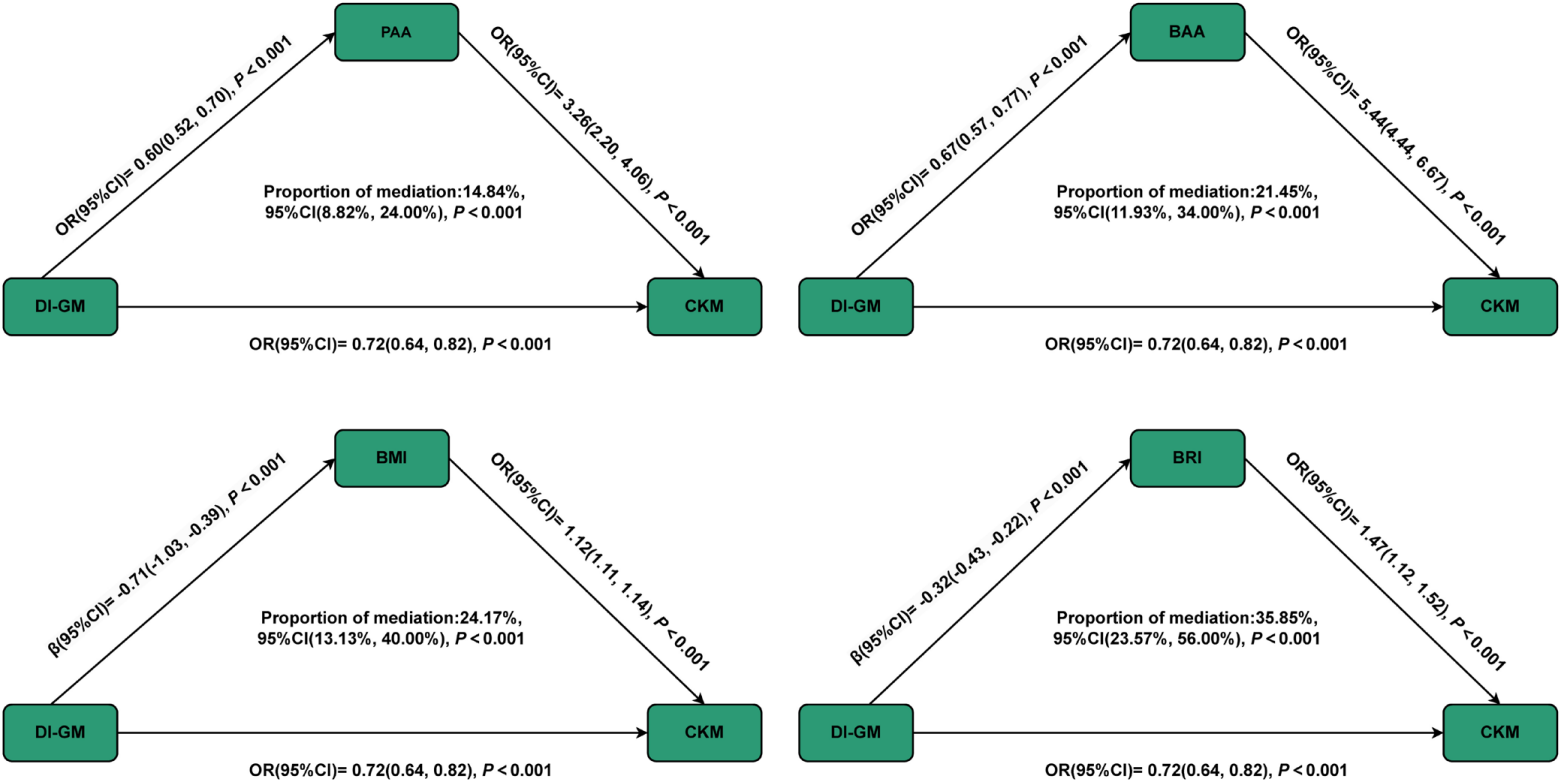
Mediation analysis of PAA, BAA, BMI, and BRI was conducted to explore their role in the relationship between DI‐GM and CKM syndrome. Models were adjusted for age, sex, marital status, education level, smoking and drinking status. DI‐GM, dietary index for gut microbiota; BMI, body mass index; BRI, body roundness index; PAA, phenotypic age acceleration; BAA, biological age acceleration; CKM, cardiovascular‐kidney‐metabolic syndrome; OR, odds ratio; CI, confidence interval.

As shown in Figure 3, higher DI‐GM is significantly associated with lower PAA (OR = 0.60, 95% CI = 0.52, 0.70) and BAA (OR = 0.67, 95% CI = 0.57, 0.77). Moreover, higher PAA (OR = 3.26, 95% CI = 2.20, 4.06) and BAA (OR = 5.44, 95% CI = 4.44, 6.67) are significantly associated with a higher risk of CKM syndrome. In the association between DI‐GM and CKM syndrome, significant mediating effects of PAA (mediated proportion (MP): 14.84%, *p* < 0.001) and BAA (MP: 21.45%, *p* < 0.001) were observed.

Furthermore, higher DI‐GM is significantly associated with lower BMI (OR: −0.71, 95% CI: −1.03, −0.39) and BRI (OR = −0.32, 95% CI = −0.43, −0.22). At the same time, higher BMI (OR = 1.12, 95% CI = 1.11, 1.14) and BRI (OR = 1.47, 95% CI = 1.12, 1.52) are significantly associated with a higher risk of CKM syndrome. In the association between DI‐GM and CKM syndrome, significant mediating effects of BMI (MP: 24.17%, *p* < 0.001) and BRI (MP: 35.85%, *p* < 0.001) were also observed.

## 4 Discussion

The study found a positive correlation between low DI‐GM and the risk of CKM syndrome. Meanwhile, PAA, BAA, BMI, and BRI significantly mediate this association.

DI‐GM is a newly proposed dietary quality index used to reflect the diversity of the gut microbiota (Zhang, Yang, et al. 2024). The gut microbiota plays a role in the occurrence and development of obesity, diabetes, CKD, and CVD by influencing a range of inflammatory pathways, neuroendocrine pathways, and immune metabolic regulatory processes (Ren et al. 2020; Tomasics et al. 2023; Usman et al. 2024; Zarate et al. 2024). The gut microbiota regulates the gene expression of many human cells. Gut metabolites, including trimethylamine N‐oxide (TMAO), lipopolysaccharides (LPS), phenylacetylglutamine (PAG), and short‐chain fatty acids (SCFAs), help prevent or reduce the formation of vulnerable plaques, thereby intervening in the progression of coronary atherosclerosis (Zheng et al. 2024). Mutations in the elastin gene (ELN) can lead to aortic valve stenosis (SVAS), thickening arterial walls (Dicks 2024). TMAO, produced by the gut microbiota, activates inflammatory NOD‐like receptors (NLRs), thereby increasing platelet formation. A randomized controlled trial (RCT) demonstrated that a healthy dietary pattern could improve cardiovascular health by altering the gut microbiota (Wang, Cross, et al. 2024). Modulating the gut microbiota may enhance the effectiveness of cardiac cell therapy (Chen et al. 2025). Research has found that the dysregulation of gut microbiota metabolites caused by specific dietary choices may contribute to the progression of vascular calcification (Cui et al. 2024). The cardiovascular health benefits observed from low red meat and Mediterranean diets can be explained by their promotion of beneficial changes in the gut microbiota environment (Ronen et al. 2024; Zambrano et al. 2024). A meta‐analysis showed increased levels of Streptococcus, Proteobacteria, and TMAO in the gut microbiota of CVD patients, while Firmicutes were reduced (Martins et al. 2024). Consistent with previous findings, low DI‐GM reflects a decrease in gut microbiota diversity, typically associated with poorer gut health and higher levels of metabolite toxicity. We found a positive correlation between low DI‐GM and the occurrence of CKM syndrome, suggesting that DI‐GM may become an effective indicator for early risk prediction of CKM syndrome.

The complex interaction between diet and aging may influence the gut microbiota, which could lead to age‐related diseases (Zhu et al. 2024). Heart failure can lead to reduced intestinal perfusion and altered gut motility, negatively affecting the gut microbiota composition. Metabolites derived from the body, released into the bloodstream, can also trigger inflammatory responses, further worsening cardiac function. Gut microbiota metabolites, including TMAO, SCFAs, and secondary bile acids (BAs), can influence gene expression processes such as DNA methylation and histone modification, mediating the progression of heart failure (Matacchione et al. 2024). The modulation of cardiovascular disease risk by gut microbiota characteristics shows differences based on age and the types of metabolic products (Garcia‐Fernandez et al. 2024; Wang, Shi, et al. 2024). There is a potential causal relationship between some gut microbiota and phenotypes that accelerate aging (Xu et al. 2024). Mouse experiments show a positive correlation between the increased relative abundance of aging‐related bacterial groups and significantly increased secondary bile acid metabolites. Multiple anti‐inflammatory metabolites are found to be at lower levels in elderly mice (Chen et al. 2024). A cross‐sectional study found that increased processed meat intake was positively correlated with BAA, while increased fiber intake was negatively correlated with BAA. BAA was positively correlated with Streptococcus and negatively correlated with unclassified Bacteroidetes and Burkholderia (Sharma et al. 2024). The microbial‐based probability of ACVD was positively correlated with age (Dong et al. 2023). BAA and PAA, as indicators of aging speed, can reflect the relationship between aging processes and health status. The relationship between low DI‐GM and the occurrence of CKM syndrome may be mediated by PAA and BAA, suggesting that the role of the gut microbiota in CKM risk may be closely related to aging and its associated diseases.

Compared to established dietary patterns, DI‐GM uniquely targets gut‐microbiota interactions through emphasizing fermented foods (e.g., yogurt, kimchi), prebiotic fibers (onions, garlic), and polyphenol‐rich foods (berries, green tea). Whereas the Mediterranean diet reduces cardiovascular risk through olive oil's monounsaturated fats (Estruch et al. 2018), and the DASH diet lowers blood pressure via sodium restriction (Appel et al. 1997), DI‐GM operates through distinct mechanisms: SCFA production, high‐fiber components (whole grains, legumes) promote butyrate synthesis, inhibiting hepatic VLDL secretion (Zhao and Zou 2023); bile acid modulation, polyphenols alter bile acid conjugation, reducing FXR signaling and triglyceride accumulation (Tveter et al. 2023); barrier enhancement, fermented foods increase mucin‐producing bacteria (Akkermansia), ameliorating endotoxemia‐related inflammation (Yue et al. 2022). This explains DI‐GM's stronger association with CKM biomarkers (e.g., cystatin C, NT‐proBNP) compared to general plant‐based patterns.

A large body of evidence confirms that obesity is a major risk factor for CVD and CKD, and is associated with a range of cardio‐renal complications. Its complex mechanisms include adipose tissue dysfunction, chronic inflammation, immune system dysregulation, and changes in the gut microbiota (Tuccinardi et al. 2024). BMI, BRI, WC, WHR, and WHtR are good indicators for predicting CVD risk (Kulkarni et al. 2023). A Chinese population cohort study showed that gut microbiota has potential in the stratification and refined management of obesity (Zeng et al. 2021). An RCT indicated that dietary prebiotics could improve gut microbiota composition, positively affecting gut‐brain signaling and higher cognitive functions, improving unhealthy eating habits and metabolic status in obese patients (Vartanian et al. 2025). Fiber and ultra‐processed food (UPF) intake significantly affect gut microbiota composition, and there are differences in the gut microbiota composition of medical students with different body mass indexes (Moreno‐Altamirano et al. 2024). SCFAs can regulate metabolic and immune signals, interrupting harmful cascades in obesity and inflammation (Kopczyńska and Kowalczyk 2024). An increase in BMI may indicate the production of different metabolites by the gut microbiota, leading to significant changes in inflammation markers, gut barrier metabolites, glucose metabolism, endocrine indicators, and fecal metabolomic profiles (Maqoud et al. 2024). Two independent cohort studies showed that changes in the gut microbiota in normal‐weight obesity (NWO) patients exacerbate the cardiovascular metabolic risk in BMI‐normal adults (Wang et al. 2025). Previous studies have shown that BMI is a key predictor of CKM syndrome and female‐specific risk enhancement factors (Zhang, Zheng, et al. 2024). In the CKM syndrome stages 0 to 33 population, a positive linear association exists between the TyG‐BMI index and increased CVD incidence (Li, Shen, et al. 2024). BMI plays a mediating role in the relationship between low DI‐GM scores and the higher risk of accelerated aging (An et al. 2024). In 65‐year‐old individuals with diabetes, the impact of obesity on the risk of cardiovascular adverse events in CKM comorbidity is more significant (Kulkarni et al. 2023). Consistent with previous research, we found that BMI and BRI mediated the relationship between low DI‐GM and the occurrence of CKM syndrome. Although mouse experiments suggest that the role of the gut microbiota in driving obesity and inflammatory responses is independent of diet and genetics (Kulkarni et al. 2023), it still suggests the necessity of further observing this relationship in human populations.

The molecular mechanisms of CKM diseases include a series of interconnected factors, such as hyperglycemia, insulin resistance, the renin‐angiotensin‐aldosterone system, advanced glycation end products, oxidative stress, lipotoxicity, endoplasmic reticulum stress, calcium handling abnormalities, mitochondrial dysfunction, impaired energy production, and chronic persistent inflammation (Sebastian et al. 2024). Personalized strategies for preventing, diagnosing, and treating CKM syndrome involving DI‐GM and the gut microbiota can be tailored according to the gut microbiota characteristics at different life stages and in various comorbid states. This includes prediction and diagnosis based on gut microbiota‐blood metabolite associations, dietary interventions, and lifestyle improvements (Shi et al. 2024).

This cross‐sectional NHANES analysis identifies associations between DI‐GM exposure and CKM syndrome but cannot establish causality. Key methodological limitations include: (1) Inability to Determine Causality/Temporal Sequence: The cross‐sectional design and simultaneous data collection preclude determining the temporal sequence between DI‐GM exposure and CKM development, leaving causation unverified. (2) Risk of Reverse Causation: Preclinical CKM (e.g., early CKD) may alter dietary behaviors (like sodium‐restricted diets), potentially causing the observed associations rather than DI‐GM causing CKM. (3) Potential for Unmeasured Confounders: Unmeasured factors, such as genetic predispositions (e.g., APOE variants) or environmental exposures (e.g., PFAS), which might jointly influence dietary choices and CKM pathways, could confound the results. (4) Limitations in Dietary Assessment: The use of self‐reported tools (like 24‐h recalls) captures only recent dietary intake, whereas CKM develops over decades. Furthermore, the single‐time assessment is susceptible to recall bias and may not reflect long‐term DI‐GM patterns. (5) Lack of Mechanistic Data: While the proposed pathway involves DI‐GM influencing CKM risk via accelerated aging/adiposity, NHANES lacks microbiome sequencing data, preventing validation of direct causal links through GM alterations. Future research directions to address these limitations should include longitudinal cohort studies tracking DI‐GM trajectories, utilizing genetic tools (e.g., mendelian randomization) to handle diet‐gene interactions, conducting large‐scale prospective multicenter studies in high‐risk groups, and incorporating paired diet and microbiome sequencing to clarify underlying mechanisms.

## 5 Conclusions

Low DI‐GM is associated with an increased prevalence of CKM. PAA, BAA, BRI, and BMI significantly mediate the relationship between DI‐GM and CKM syndrome. Given the close association between diet, gut microbiota, and CKM syndrome, future risk prediction and dietary interventions for CKM patients should consider the clinical predictive value of DI‐GM.

## Author Contributions

Conceptualization: Dongdong Yu, Ting Cheng, Zehuai Wen. Data curation: Dongdong Yu, Ting Cheng. Formal analysis: Dongdong Yu, Ting Cheng. Funding acquisition: Zehuai Wen. Methodology: Dongdong Yu, Ting Cheng. Software: Dongdong Yu. Supervision: Zehuai Wen. Validation: Geng Li, Li Zhou, Yuxuan Shi, Xingying Qiu. Visualization: Yuxuan Shi, Yue Yang, Xingying Qiu. Writing – original draft: Dongdong Yu, Ting Cheng. Writing – review and editing: Zehuai Wen.

## Ethics Statement

The research protocol has been approved by the Research Ethics Review Committee of the National Center for Health Statistics (NCHS). All participants provided written consent at the time of recruitment. As this study is a retrospective analysis and poses no risk of exposure to personally identifiable information, no additional ethical review or informed consent is required.

## Conflicts of Interest

The authors declare no conflicts of interest.

## Supporting information


**Data S1:** Supporting Information.

## Data Availability

Data Availability StatementThe data for this study can be obtained from the NHANES official website (https://wwwn.cdc.gov/nchs/nhanes/Default.aspx).
