# Supplementary material for: Association of the Dietary Index for Gut Microbiota and Cardiovascular‐Kidney‐Metabolic Syndrome: The Mediation Effect of Phenotypic and Biological Age Acceleration, BMI, and BRI
Source: Food Sci Nutr. 2025 Sep 7;13(9):e70815. doi: 10.1002/fsn3.70815 (PMC12415064; doi:10.1002/fsn3.70815)
Supplement: Supplementary file 1 — Data S1: Supporting Information. [file FSN3-13-e70815-s001.docx]

**Supplement 1**

**Supplementary Methods**

**1. Definition of DI-GM**

| Components of  DI-GM | Food items included in NHANES | Scoring criteria |
| --- | --- | --- |
| Beneficial to gut microbiota | Avocados, Broccoli, Chickpeas, Coffee, Cranberries, Fermented dairy (including yogurt, cheese, kefir, sour cream, buttermilk), Fiber, Soybean (including Soy milk, Tofu), Whole grains | Score 1 - Consumption ≥ sex-specific median  Score 0 - Otherwise |
| Unfavorable to gut microbiota | Refined grains, Processed meat, Red meat | Score 0 - Consumption ≥ sex-specific median  Score 1 - Otherwise |
|  | High-fat diet (% energy) | Score 0 - Consumption ≥ 40%  Score 1 - Otherwise |

**2. Cardiovascular-Kidney-Metabolic (CKM) Syndrome Staging Criteria**

The staging criteria for CKM syndrome are directly adapted from the supplementary methods (eAppendix 1) of Aggarwal et al., as published in JAMA (doi:10.1001/jama.2024.6892, PMCID: PMC11079779, PMID: 38717747). This framework operationalizes the 2023 American Heart Association (AHA) guidelines and was validated using NHANES data. The criteria are reproduced below to ensure consistency and comparability with prior research, leveraging the publicly accessible methodology detailed in the original work. As the publication is freely available via JAMA and PubMed Central, formal permission was not sought; instead, this reuse is acknowledged under standard academic practices for transparent replication of validated frameworks. The CKM syndrome stages are defined as follows, with thresholds and risk factors explicitly based on Aggarwal et al.'s eAppendix 1:

CKM syndrome stages were identified among NHANES participants in accordance with the 2023 American Heart Association (AHA) Presidential Advisory on CKM Health. We only included adults ≥20 years in the fasting subsample of NHANES, which is a nationally representative subsample of the US noninstitutionalized population and comprises approximately half of NHANES. Among patients in the fasting subsample, missingness rates for all physical examination and laboratory measurements were low (<10%). Thus, no correction for missingness was performed. Participants with insufficient data to determine CKM stage were excluded (<5%).

In this analysis, CKM stage definitions were adapted to data available in NHANES. Age, sex, race, and ethnicity were determined by self-report. Diabetes and hypertension definitions were based on biomarkers (e.g., glycated hemoglobin) or healthcare diagnoses, while history of CVD (heart failure, coronary heart disease, and stroke) was determined by self-report. Estimated glomerular filtration (GFR) rate was calculated using the race-free CKD-EPI 2021 creatinine equation.

CKM Stage 0 included participants with normal body mass index (BMI) (<23 kg/m2 for individuals with Asian ethnicity and <25 kg/m2 for other racial and ethnic groups), normal waist circumference (<80 and <90 cm for women and men with Asian race, respectively, and <88 and <102 cm for women and men in all other race and ethnicity categories, respectively) who did not meet criteria for the other stages.

CKM Stage 1 identified individuals with an elevated BMI (≥23 kg/m2 for individuals with Asian race and >25 kg/m2 for all other race and ethnic groups), an elevated waist circumference (≥80 and ≥90 cm for women and men with Asian race, respectively, and ≥88 and ≥102 cm for women and men in other race and ethnicity categories, respectively), or prediabetes (defined as a glycated hemoglobin of 5.7% to <6.5% or a fasting blood glucose of 100 mg/dL to <126 mg/dL).

CKM Stage 2 identified participants with metabolic risk factors or moderate-to-high-risk CKD per Kidney Disease Improving Global Outcomes (KDIGO) criteria, as recommended by the AHA.1 Qualifying metabolic risk factors included elevated fasting serum triglycerides (≥135 mg/dL), hypertension, diabetes, or metabolic syndrome (≥3 of the following: elevated waist circumference, low high density lipoprotein cholesterol (HDL) level [<40 mg/dL or <50 mg/dL for men or women, respectively], fasting serum triglycerides ≥150 mg/dL, elevated blood pressure [systolic blood pressure ≥130, diastolic blood pressure ≥80 mmHg, and/or use of blood pressure-lowering medications], or prediabetes). CKD stages were identified based on GFR and urinary albumin-to-creatinine ratio.

CKM Stage 3 was identified based of the presence of very-high-risk KDIGO CKD stages1 or a high-predicted 10-year CVD risk. 10-year cardiovascular risk was estimated with the AHA Predicting Risk of CVD EVENTs (PREVENT) equations.3 High risk was defined as ≥20% 10-year CVD risk (based on recommended thresholds [https://professional.heart.org/en/guidelines-and-statements/prevent-calculator]). The PREVENT equations were developed and validated for adults 30-79 years of age. As such, risk was not estimated for adults <30 years. However, to minimize underestimation of CKD Stage 3, adults ≥80 years were not excluded from 10-year CVD risk. Instead, adults ≥80 years were assigned an age of 79 years when determining 10-year CVD risk to allow for conservative estimates. Further, PREVENT was developed for variables with the following ranges: total cholesterol 130-320 mg/dL, HDL 20-100 mg/dL, systolic blood pressure 90-200 mmHg, and GFR 14-140 mL/min/1.73m². To approximate PREVENT risk strata, values for these variables above or below these bounds were set to the upper or lower bounds of allowable values respectively (for example, total cholesterol of 330 mg/dL was set as 320 mg/dL). Cardiac biomarkers and cardiovascular imaging were not available to identify subclinical CVD.

CKM Stage 4 was identified based on self-reported established cardiovascular disease (coronary heart disease, angina, heart attack, heart failure, and stroke). Atrial fibrillation and peripheral artery disease were not included, as these data were not available.

References:

[1] NDUMELE C E, RANGASWAMI J, CHOW S L, et al. Cardiovascular-Kidney-Metabolic Health: A Presidential Advisory from the American Heart Association [J]. Circulation, 2023, 148(20): 1606-1635.

[2] INKER L A, ENEANYA N D, CORESH J, et al. New Creatinine- and Cystatin C-Based Equations to Estimate GFR without Race [J]. N Engl J Med, 2021, 385(19): 1737-1749.

[3] KHAN S S, MATSUSHITA K, SANG Y, et al. Development and Validation of the American Heart Association's PREVENT Equations [J]. Circulation, 2024, 149(6): 430-449.

[4] AGGARWAL R, OSTROMINSKI J W, VADUGANATHAN M. Prevalence of Cardiovascular-Kidney-Metabolic Syndrome Stages in US Adults, 2011-2020 [J]. JAMA, 2024, 331(21): 1858-1860.

**3.** **Biological age (BioAge)**

The Biological age algorithm is derived from a series of regressions of individual biomarkers on Chronological Age (CA) in a reference population. The equation takes information from *n* number of regression lines of CA regressed on *n* biomarkers ^[1]^. The formula is:

$$BioAge=\frac{\sum_{i=1}^{n} \left( x_{i}-q_{i} \right)\frac{k_{i}}{s_{i}^{2}}+\frac{Chronological Age}{s_{BA}^{2}}}{\sum_{i=1}^{n} {(\frac{k_{i}}{s_{i}})}^{2}+\frac{1}{s_{BA}^{2}}}$$

Where *x* is the value of biomarker *i* measured for an individual. For each biomarker *i*, the parameters *k*, *q*, and *s* are estimated from a regression of CA on the biomarker in the reference sample. The *q*, *k*, and *s* are the regression intercept, slope, and root mean squared error, respectively. *s_BA_* is a scaling factor equal to the square root of the variance in CA explained by the biomarker set in the reference sample. In the ‘BioAge’ package, the reference sample is NHANES III non-pregnant participants. Algorithm parameters are estimated separately for male and female.

**4.** **Phenotypic age (PhenoAge)**

The PhenoAge algorithm is derived from multivariate analysis of mortality hazards. The original PhenoAge algorithm was constructed from elastic-net Gompertz regression of mortality on 42 biomarkers in the NHANES III ^[2]^. This analysis selected nine biomarkers described above and CA. The formula is:

$$PhenoAge=141.8254+\frac{\ln\left[ -0.005807831\times ln \left( 1-M \right) \right]}{0.08742185}$$

$$M=1-exp(\frac{-1.386933\times exp(xb)}{0.007250078})$$

$$xb=-18.264084479-0.037550125\times albumin\_gL+0.001977458\times Alkaline Phosphatase+ 0.178246261\times ln(CRP)-0.001046473\times Total cholesterol+0.846406370\times ln (Creatinine) +0.161423930\times HbA1c+0.007376810\times Systolic blood pressure-0.013646067\times Blood urea nitrogen+0.049875933\times Uric acid-0.010733596\times Lymphocyte Percent+0.021307220\times Mean Cell Volume+0.062521994\times White Blood Cell Count+0.078026776\times Chronological Age$$

**References:**

1. Kwon D, Belsky DW. A toolkit for quantification of biological age from blood chemistry and organ function test data: BioAge. Geroscience 2021, 43(6):2795-2808.

2. Levine ME, Lu AT, Quach A, et al. An epigenetic biomarker of aging for lifespan and healthspan. Aging (Albany NY). 2018;10(4):573-591.

**Supplement 2**

**Table S1. Sensitivity Analysis for Continuous Variables Using Non-parametric Methods**

| Variable | total | Non-CKM (*n*= 2042) | CKM (*n*=5210) | *P* |
| --- | --- | --- | --- | --- |
| Age | 48.00(34.00,60.00) | 36.00(26.00,48.00) | 53.00(40.00,64.00) | < 0.001 |
| BMI | 27.90(24.26,32.65) | 25.30(22.50,28.95) | 29.39(25.65,33.90) | < 0.001 |
| BRI | 4.96(3.76,6.52) | 3.90(2.91,5.06) | 5.50(4.34,7.15) | < 0.0001 |
| PIR | 3.10(1.54,5.00) | 3.32(1.66,5.00) | 3.00(1.49,5.00) | 0.17 |
| PA | 39.70(25.90,53.25) | 26.27(17.20,39.22) | 45.72(33.49,58.26) | < 0.001 |
| BA | 28.46(7.59,46.73) | 21.01(-1.95,32.16) | 35.61(11.11,50.89) | < 0.001 |

BMI: body mass index; BRI: body roundness index; PIR: poverty income ratio; PA: phenotypic age; BA: biological age; CKM: cardiovascular-kidney-metabolic syndrome.

**Table S2. Univariate regression results**

| Variable | OR(95% CI) | *P* |
| --- | --- | --- |
| Age | 1.06(1.05,1.07) | < 0.001 |
| Sex |  |  |
| Male | ref | ref |
| Female | 0.67(0.57,0.77) | < 0.001 |
| BMI | 1.12(1.10,1.14) | < 0.001 |
| BRI | 1.55(1.46,1.65) | < 0.001 |
| DI-GM | 0.92(0.88,0.96) | < 0.001 |
| Race/Ethnicity |  |  |
| White | ref | ref |
| Black | 0.97(0.81,1.16) | 0.70 |
| Mexican | 0.97(0.78,1.23) | 0.85 |
| Other | 0.95(0.77,1.18) | 0.63 |
| Marital status |  |  |
| married_partner | ref | ref |
| alone | 0.79(0.68,0.93) | 0.006 |
| Education |  |  |
| Less than high school | ref | ref |
| High school or equivalent | 0.54(0.40,0.75) | < 0.001 |
| College or above | 0.37(0.26,0.53) | < 0.001 |
| Smoke |  |  |
| no | ref | ref |
| yes | 1.48(1.26,1.74) | < 0.001 |
| Alcohol |  |  |
| no | ref | ref |
| yes | 0.73(0.56,0.94) | 0.020 |
| PIR | 0.96(0.91,1.02) | 0.190 |
| PAA |  |  |
| youth | ref | ref |
| old | 3.08(2.34,4.06) | < 0.001 |
| BAA |  |  |
| youth | ref | ref |
| old | 1.83(1.37,2.44) | < 0.001 |
| DI-GM: dietary index for gut microbiota; BMI: body mass index; BRI: body roundness index; PIR: poverty income ratio; PAA: phenotypic age acceleration; BAA: biological age acceleration; OR: odds ratio; CI: confidence interval; ref: reference level/category. | | |

**Table S3. Racial Sensitivity Analysis in Multivariable Regression for DI-GM and CKM Syndrome Association**

|  | Crude model | |  | Model 1 | |  | Model 2 | |  | Model 3 | |
| --- | --- | --- | --- | --- | --- | --- | --- | --- | --- | --- | --- |
| Character | OR(95%CI) | *P* |  | OR(95%CI) | *P* |  | OR(95%CI) | *P* |  | OR(95%CI) | *P* |
| DI-GM | 0.92(0.88,0.96) | <0.001 |  | 0.87(0.83,0.92) | <0.001 |  | 0.91(0.87,0.96) | <0.001 |  | 0.92(0.87,0.97) | 0.002 |
| DI-GM category |  |  |  |  |  |  |  |  |  |  |  |
| Higher DI-GM | ref |  |  | ref |  |  | ref |  |  | ref |  |
| Lower DI-GM | 1.27(1.09,1.49) | 0.003 |  | 1.50(1.24,1.82) | < 0.001 |  | 1.36(1.11,1.67) | 0.003 |  | 1.34(1.09,1.63) | 0.010 |
| DI-GM: dietary index for gut microbiota; BMI: body mass index; BRI: body roundness index; PIR: poverty income ratio; PAA: phenotypic age acceleration; BAA: biological age acceleration; OR: odds ratio; CI: confidence interval.  Crude model: adjusted for none; ref: reference level/category.  Model 1: adjusted for age, sex, marital status, education.  Model 2: adjusted for age, sex, marital status, education, smoke, alcohol, BMI, BRI.  Model 3: adjusted for age, sex, marital status, education, smoke, alcohol, BMI, BRI, PAA, BAA, Race/Ethnicity. | | | | | | | | | | | |

**Table S4. Subgroup Analysis of DI-GM and CKM Syndrome Risk**

| Character | Higher DI-GM | Lower DI-GM | *P* | *P* for interaction |
| --- | --- | --- | --- | --- |
| Sex |  |  |  | 0.348 |
| Male | ref | 1.44(1.06,1.95) | 0.020 |  |
| Female | ref | 1.26(0.95,1.68) | 0.105 |  |
| Marital status |  |  |  | 0.730 |
| Couple | ref | 1.37(1.05,1.77) | 0.021 |  |
| Alone | ref | 1.29(0.89,1.88) | 0.174 |  |
| Education |  |  |  | 0.399 |
| Less than high school | ref | 0.87(0.41,1.87) | 0.718 |  |
| High school or equivalent | ref | 1.35(1.07,1.70) | 0.012 |  |
| College or above | ref | 1.37(1.05,1.78) | 0.020 |  |
| Smoke |  |  |  | 0.317 |
| No | ref | 1.45(1.12,1.90) | 0.007 |  |
| Yes | ref | 1.21(0.90,1.64) | 0.199 |  |
| Alcohol |  |  |  | 0.752 |
| No | ref | 1.30(0.56,3.03) | 0.534 |  |
| Yes | ref | 1.35(1.10,1.65) | 0.005 |  |

**Table S5. Associations between DI-GM and CKM using the IPTW method**

|  | Crude model | |  | Model 1 | |  | Model 2 | |  | Model 3 | |
| --- | --- | --- | --- | --- | --- | --- | --- | --- | --- | --- | --- |
| Character | OR(95%CI) | *P* |  | OR(95%CI) | *P* |  | OR(95%CI) | *P* |  | OR(95%CI) | *P* |
| DI-GM category |  |  |  |  |  |  |  |  |  |  |  |
| Higher DI-GM | ref |  |  | ref |  |  | ref |  |  | ref |  |
| Lower DI-GM | 1.10(0.94,1.28) | 0.25 |  | 1.15(1.03,1.34) | 0.04 |  | 1.17(1.02,1.35) | 0.03 |  | 1.15(1.01,1.33) | 0.04 |
| DI-GM: dietary index for gut microbiota; BMI: body mass index; BRI: body roundness index; PIR: poverty income ratio; PAA: phenotypic age acceleration; BAA: biological age acceleration; OR: odds ratio; CI: confidence interval; IPTW: inverse probability of treatment weighting.  Crude model: adjusted for none; ref: reference level/category.  Model 1: adjusted for age, sex, marital status, education.  Model 2: adjusted for age, sex, marital status, education, smoke, alcohol, BMI, BRI.  Model 3: adjusted for age, sex, marital status, education, smoke, alcohol, BMI, BRI, PAA, BAA. | | | | | | | | | | | |

**Supplement 3**


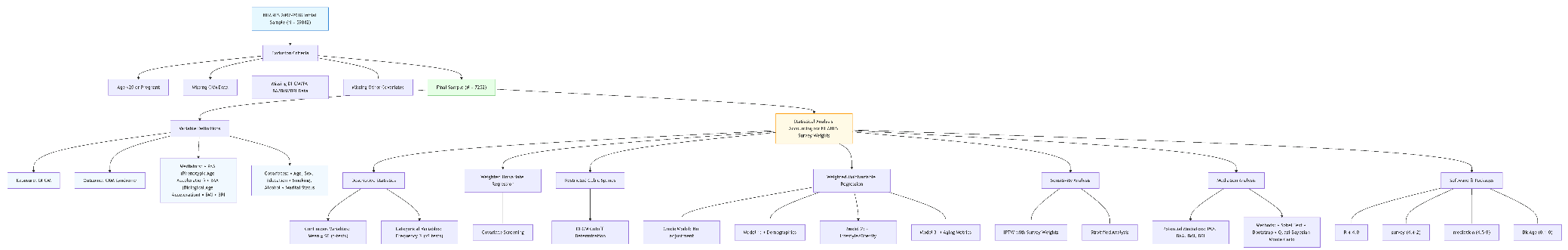


Figure S1 Statistical analysis flowchart. DI-GM: dietary index for gut microbiota; BMI: body mass index; BRI: body roundness index; PAA: phenotypic age acceleration; BAA: biological age acceleration; CI: confidence interval; IPTW: inverse probability of treatment weighting.


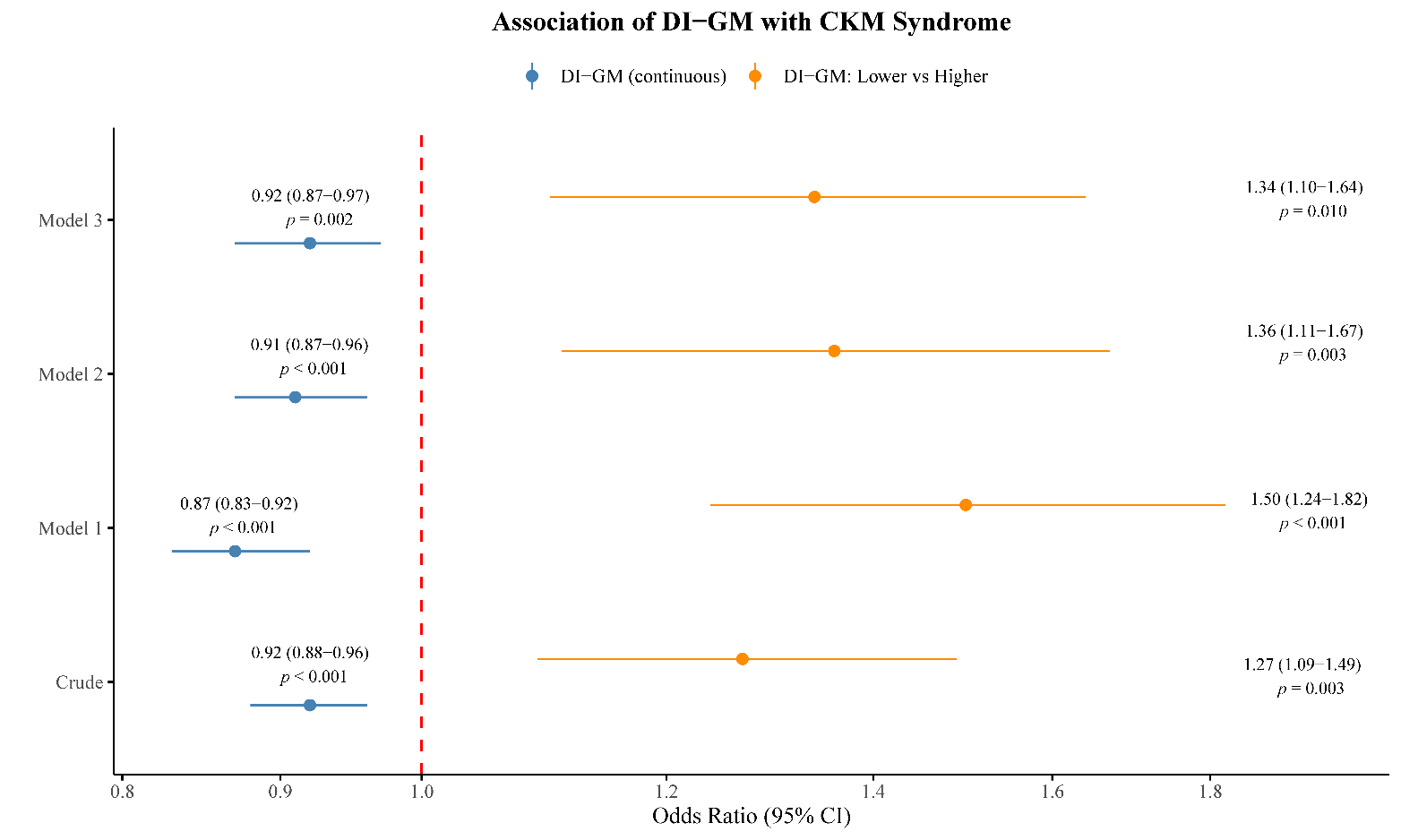


Figure S2 Forest Plot of DI−GM Associations with CKM Syndrome. DI-GM: dietary index for gut microbiota; BMI: body mass index; BRI: body roundness index; PIR: poverty income ratio; PAA: phenotypic age acceleration; BAA: biological age acceleration; OR: odds ratio; CI: confidence interval; IPTW: inverse probability of treatment weighting. Crude model: adjusted for none; ref: reference level/category. Model 1: adjusted for age, sex, marital status, education. Model 2: adjusted for age, sex, marital status, education, smoke, alcohol, BMI, BRI. Model 3: adjusted for age, sex, marital status, education, smoke, alcohol, BMI, BRI, PAA, BAA.
